# Infrared and Visible Image Fusion Based on Different Constraints in the Non-Subsampled Shearlet Transform Domain

**DOI:** 10.3390/s18041169

**Published:** 2018-04-11

**Authors:** Yan Huang, Duyan Bi, Dongpeng Wu

**Affiliations:** 1 Aeronautics and Astronautics Engineering College, Air Force Engineering University, Xi’an 710038, Shaanxi, China; biduyan@126.com; 2 School of Management Engineering, Xi’an University of Finance and Economics, Xi’an 710100, Shaanxi, China; 3 The 93575 Unit of PLA, Chengde 067000, Hebei, China; wdp_image@126.com

**Keywords:** image fusion, constraints, infrared and visible images, details of image, salient targets, Nash equilibrium

## Abstract

There are many artificial parameters when fuse infrared and visible images, to overcome the lack of detail in the fusion image because of the artifacts, a novel fusion algorithm for infrared and visible images that is based on different constraints in non-subsampled shearlet transform (NSST) domain is proposed. There are high bands and low bands of images that are decomposed by the NSST. After analyzing the characters of the bands, fusing the high level bands by the gradient constraint, the fused image can obtain more details; fusing the low bands by the constraint of saliency in the images, the targets are more salient. Before the inverse NSST, the Nash equilibrium is used to update the coefficient. The fused images and the quantitative results demonstrate that our method is more effective in reserving details and highlighting the targets when compared with other state-of-the-art methods.

## 1. Introduction

Image fusion plays a very important role in many fields. In remote sensing [1], multispectral images and panchromatic images are integrated into an image; the fused image can obtain quality spatial resolution and the quality spectral resolution. In medical imaging [2], the MRI images and PET images are fused to help diagnose the condition. In modern military technologies [3,4], the fusion of thermal infrared (IR) images and visible images is becoming more important for night action. In this paper, we concentrate on the fusion of IR images and visible images, which can lead to better performance for target recognition and human visual perception. 

The goal of IR and visible image fusion is to integrate them into an image, which can contain most information in the source images without distortion or loss. The visible spectrum is better in spatial resolution, but it is easily affected by illumination and disguise. In contrast, IR images are not affected by illumination and disguise, but do suffer form low resolution. Therefore, integrating the two types of images can be beneficial for target detection and recognition at night.

The image fusion algorithm can be divided into two categories. One type is fusion algorithms, which achieve in the spatial domain, such as the weight method, pyramid transform method [5], principal component analysis (PCA) [6], saliency method [7], and so on. Those algorithms are simple to implement, and they can obtain some useful images, but the fused images lose information on occasion sometimes. Another type of fusion algorithms fuse images in the transform domain. Chao [8] fused images based on wavelets in 2004, in the high- and low-level bands of the fused the images, respectively. The result is effective, but loses some orientation information in the image. Due to the insufficient orientations in wavelets, Do [9] proposed the contourlet transform based on wavelets. Wang [10] fused IR images and visible images based on the contourlet transform, and the fused images perform well with respect to the image details, but with sampling, there are Gibbs phenomena in the final images. Thus, the non-subsampled contourlet transform is used in the fusion algorithm [11], overcoming the shortcoming of the Gibbs phenomenon. Guo [12,13,14] proposed the shearlet transform in 2008, and it not only possessed the above properties, but it is equipped with a rich mathematical structure that is associated to multi-resolution analyses, and it forms a tight frame at various levels and directions, reducing the complexity and improving the efficiency of the operation. Wang [15] proposed a novel fusion algorithm that is based on improved shearlet transform to enhance the quality of the fused image. Wan [16] proposed a method based on the guided filter and improved sum-modified-Laplacian (SML) in the non-subsampled shearlet transform (NSST) domain. However, most of the above algorithms set a large number of artificial parameters, and there are many artifacts in the final results that seriously affect the target edge and the background information.

Ma [17] proposed an infrared and visible image fusion algorithm that is based on the total variation, the optimal image is calculated by iterations, and the details in the visible image can be retained as much as possible, while also maintaining the target characters in the IR image. However, the fusion results look like an enhanced IR image, which is not suitable for many applications. To improve the quality of the fused image, we proposed a novel fusion algorithm for IR images and visible images. On one hand, the details in the visible image can be reserved; on the other hand, the salient target and some important information are transformed in the final image. The algorithm fuses images that are based on the NSST, but uses the gradient and saliency constraints in the fusion rules.

The contributions of this paper are as follows: we take advantage of the total variation minimization to optimize the fusion image based on the NSST domain, and both details and target information are well reserved. In addition, we provide both qualitative and quantitative comparisons with several state-of-the-art algorithms on a publicly available dataset. When compared to those algorithms, our results are more suitable for human vision systems and processing, and thus, improve the reliability of automatic target detection and recognition systems.

The rest of the paper is organized as follows: Section 2 introduces the related NSST, and, at the same time, we establish the constraints based on the gradient and the saliency of the rules of different levels. In Section 3, we compare our approach with several of the most advanced methods. Section 4 summarizes the full text. 

## 2. Methods

### 2.1. Preliminaries 

When *n* = 2, the affine systems with composite dilations are defined as follows [10,11]:

(1) $$\left\{\psi_{j,l,k}(x)={\left|\det B\right|}^{j/2}\psi (A^{l}B^{j}x-k):l,j\in Z,k\in Z^{2}\right\}$$

where $\psi \in L^{2}(R^{2})$, *A* and *B* are 2 × 2 invertible matrices with det(*B*) = 1, *A* and *B* control the level and direction of the transform, respectively, and *j*, *l* and *k* are the parameters that control the levels, directions, and transform, respectively. The values of the two matrices play an important role in the transform, and the values of *A* and *B* for the shearlet transform are defined as follows:

(2) $$A=\left(\begin{array}{cc} a & 0\\ 0 & \sqrt{a} \end{array}\right),B=\left(\begin{array}{cc} 1 & s\\ 0 & 1 \end{array}\right)$$

where *a* = 4 and *s* = 1 in the Equation (2).

When processing the digital image using the shearlet transform, we need the discrete shearlet transform. There are subsamples in the operation of discretization, so there is the impossibility of Gibbs phenomenon in the images. To overcome the shortage, the NSST is proposed [14].

The NSST can be more sparse and effective when simulating the images, which are two-dimensional, and it can be considered as the optimal tool to analyze two-dimensional data. There are also some methods of image fusion that are based on the NSST in recent years, which can reserve the characteristics of different frequencies, but the universal fusion rule relies on artificial parameters, which leads to the artifacts. Therefore, this paper proposes a fusion rule that is based on different constraints in the NSST domain to improve the quality of the fused image.

### 2.2. Proposed Method

#### 2.2.1. Model of Image Fusion

The model of the traditional infrared and visible image fusion is mainly expressed as Equation (3):

(3) $$\tilde{f}=\alpha \cdot I+\beta \cdot V$$

In Equation (3), $\tilde{f}$ represents the fused image, α and β represent the weight factors, respectively, I represents the infrared image, and V represents the visible image. In general, the setting of the weight factors needs to be man-made. Although this approach is convenient, the quality of the fused image is not optimal, and results in artifacts. Thus, in this paper, we assume the optimal fused image is f. The relationship between the optimal fused image and the original images can be expressed by Equation (4):

(4) $$\min\int {\left\|f-\alpha \cdot I-\beta \cdot V\right\|}_{2}^{2}$$

Equation (4) is the fidelity constraint of the fused image, the result is similar to the fused image by Equation (3), rely on the Equation (4) only, and cannot avoid the existence of artifacts. Therefore, in different frequency bands, we build more constraints for different frequency bands, according to their characteristics.

#### 2.2.2. Strategy for High Frequency Coefficients

The low-frequency coefficient *f_d_* and the high-frequency coefficient group {*f_g_*} can be obtained by an image *f* transform by the NSST. The high-frequency coefficients contain a great deal of detailed information [18] and the regularization term of the gradient constraint is used in our method to preserve the information better:

(5) $$\nabla_{i}f=\sqrt{{\left(\nabla_{i}^{h}f\right)}^{2}+{\left(\nabla_{i}^{v}f\right)}^{2}}$$

As Equation (5) shows, $\nabla_{i}f$ represent the gradient value of pixel *i* in the image *f*. $\nabla_{i}^{h}f=f_{i}-f_{r(i)}$ and $\nabla_{i}^{v}f=f_{i}-f_{b(i)}$ represent the neighbor to the right and below pixel *i*. When pixel *i* is located in the edges, the value of its gradient is large; on the other hand, when it is located in the smooth area, the value is small. The *f*_(*i*)_ represents the value of the image in the pixel *i*, *f_r_*_(*i*)_ represents the value of infrared image in the pixel *i*, and *f_r_*_(*i*)_ represents the value of the visible image in the pixel *i*.

After the fusion, the fused image should preserve the advantage of the gradient in the infrared image or visible image as much as possible. If the gradients of some area in the different images are small, then the area should be inhibited because of the little information that is contained in this area. 

As Ma [19] mentioned in the paper, they constrained the fused image to have a similar gradient with the visible image, as the equation below:

(6) $$\min\int {\left\|\nabla f-\nabla I\right\|}_{0}$$

**Figure 1:**
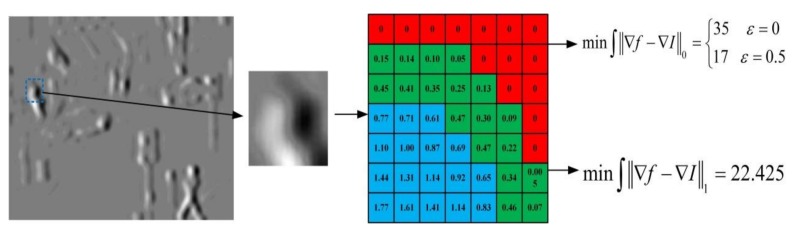
The illustration of two type of norm.

However, in infrared images, there is still some useful information for the gradient, so we constrain the gradient of the fused image *f* as:

(7) $$\min\int {\left\|\nabla f_{g}-W_{I}\cdot \nabla I_{g}-W_{V}\cdot \nabla V_{g}\right\|}_{1}$$

where the $\nabla f_{g}$ is the gradient of the high-level frequency that should be solved, $\nabla I_{g}$ and $\nabla V_{g}$ are the gradients of the original images respectively, and *W_I_* and *W_V_* are the weights for the infrared image and the visible image, respectively. It is widely known that the *l^0^* norm is difficult to solve, and the *l*^1^ norm encourages sparsity, thus, we consider minimizing the constraints that use the *l*^1^ norm as superior. We compare the *l*^0^ norm and the *l*^1^ norm, and the result is shown in Figure 1.

Figure 1 shows the high-frequency coefficients in the first iteration of the algorithm implementation in this paper, analyzing the data in the blue frame area. The *l*^0^ norm constraint should theoretically be 35, but the actual operation needs to set the threshold ε in order to determine its value. If ε = 0.5, then the value in the green frame area should be 0, and the value in the blue area will be smaller when ε is reduced. The *l*^1^ norm is constrained directly by the absolute value, sparseness is lacking, but more precise, and the calculation is simple. Thus, we choose the two coefficients of the *l*^1^ norm constraint.

The weights of the two source images are weighted, and the specific weights are set as follows:

(8) $$W_{I}^{i}=\frac{\nabla I_{g}^{i}}{\sum_{i\in N}\left(\nabla I_{g}^{i}+\nabla V_{g}^{i}\right)}$$

Equation (8) represents the weight value at pixel *i*, N represents the number of pixels adjacent to *i*, and we select a smaller 3 × 3 pixel block for the weight calculation. By the weighting of Equation (8), the gradient difference is increased and the calculation is more favorable.

The fusion rules of the high frequency part can be set by the minimization cost function of Equation (9):

(9) $$E_{g}=\int {\left\|f_{g}-\alpha \cdot I_{g}-\beta \cdot V_{g}\right\|}_{2}^{2}+\int {\left\|\nabla f_{g}-W_{I}\cdot \nabla I_{g}-W_{V}\cdot \nabla V_{g}\right\|}_{1}$$

In Equation (9), the unknown parameters have the optimal fusion *f_g_* and the weight parameters *α* and *β*, which is an unsolvable problem. To simplify the solution, we set the relationship between the parameters α and *β* and update Equation (9):

(10) $$E_{g}=\int {\left\|f_{g}-\alpha \cdot I_{g}-(1-\alpha )\cdot V_{g}\right\|}_{2}^{2}+\int {\left\|\nabla f_{g}-W_{I}\cdot \nabla I_{g}-W_{V}\cdot \nabla V_{g}\right\|}_{1}$$

Equation (10) can be transformed into a classical total variation equation for solving. We assume that $Z=f_{g}-W_{I}\cdot I_{g}-W_{V}\cdot V_{g}$ and Equation (10) can be transformed, as follows:

(11) $$E_{g}=\int {\left\|Z+W_{I}\cdot I_{g}+W_{V}\cdot V_{g}-\alpha \cdot I_{g}-(1-\alpha )\cdot V_{g}\right\|}_{2}^{2}+\int {\left\|\nabla Z\right\|}_{1}$$

Discretizing Equation (11), we obtain the final formula [20]:

(12) $$E_{g}=\sum_{\Omega }{\left(Z+W_{I}\cdot I_{g}+W_{V}\cdot V_{g}-\alpha \cdot I_{g}-(1-\alpha )\cdot V_{g}\right)}^{2}+\sum_{\Omega }\left|\nabla Z\right|$$

where Ω represents the entire image field. Calculating the value of *Z*, the optimal high-frequency transform coefficients are obtained by transformation.

### 2.3. Strategy for Low-Frequency Coefficients

**Figure 2:**
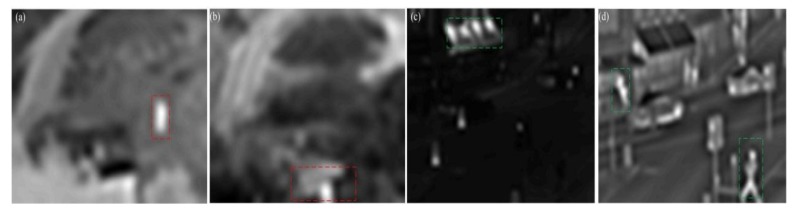
The salient target in the low level coefficient. (**a**) The low-frequency band of the infrared image for the first group; (**b**) the low-frequency band of the visible image for the first group; (**c**) the low-frequency band of the infrared image for the second group; and (**d**) the low-frequency band of the visible image for the second group.

In the low-frequency coefficient, the main energy of the image is concentrated, and the subject of the image is in the low-frequency coefficient. Figure 2 shows the low frequency coefficients of multiple images, and different salient targets are embodied in different sensor images. 

Figure 2 shows the two groups of low-frequency coefficients of the image, and the salient objects in Figure 2a,b are the thermal objects. As indicated by the red box in Figure 2a, the salient object of the main target in Figure 2b is the roof. The significance of the target in the infrared image is more obvious, the significance of the latter set of images includes heat source objects and light reflection text, and, in Figure 2c,d is the performance of the green box content. Thus, the key to the fusion of low-frequency coefficients is to optimize the performance of the significant targets. 

To improve the salience of the fused image, we constrain the low-frequency coefficients after fusing. Firstly, the salient extraction of low-frequency coefficients is analyzed below.

The extraction of salient targets has been extensively studied. In 1998, Itti first proposed a salient detection method that was based on human vision [21], identified salient areas based on a variety of a priori knowledge of the visual mechanism, but the results were not accurate enough. Additionally, many scholars have proposed new detection methods. In 2007, Tie [22] proposed the method of using a random field model based on Itti’s model, where the global information, local information, and scale information were taken into account. In 2011, Xie [23] proposed a salient detection method based on the Bayesian model, the result is suitable for simple backgrounds, but there is noise with the complex background. In 2012, Stas [24] determined the salient areas based on neighborhood relationships, obtaining better results with the context information. In 2014, Yang [25] used the propagation map to obtain salient graphs, and was able to extract the main target. In 2016, Sun [26] used multi-regional mergers to obtain salient areas, thus improving the accuracy of the test. These algorithms have achieved good results in detecting, but these algorithms are computationally complex. In our method, we use the gray image as the experimental images, so we need to concisely calculate the salience of the low-frequency coefficients. A salient area is determined by using the prior image contrast:

(13) $$P_{X}^{i}=e^{\left|X(i)\right|/\sum_{j\in \omega }\left|X(j)\right|}$$

In Equation (13), $P_{X}^{i}$ represents the value of pixel *i* in the image *X*, *X*(*i*) represents the gray value of the pixel *i*, and *ω* represents the adjacent area of pixel *i*. As Equation (13) shows, the value of salience is higher in the pixels whose contrast is higher. However, we can obtain the single value of the pixel in the image; for the target, we need to detect the salient area:

(14) $$S_{X}^{i}=\frac{P_{X}^{m}}{L_{dis}(X(i),X(m))+L_{val}(X(i),X(m))}$$

(15) $$P_{X}^{m}=\max(P_{X}^{\omega },i\in \omega )$$

where *L_dis_* and *L_val_* represent the geometric distance and gray value distance between the two pixels. $S_{X}^{i}$ represents the salient value of pixel *i* in image *X*. As Equation (15) shows, $P_{X}^{m}$ represents the extreme values in area *ω*. The closer to the extremes of the salient point, the value of salience is higher. The salience of the low-frequency portion can be extracted by Equations (13)–(15).

We fuse the low-frequency coefficient with the salient constraints, as follows:

(16) $$\min\int {\left\|S_{f}-S_{c}\right\|}_{1}$$

where *S_f_* and *S_c_* represent the salient values of the fused low-frequency coefficient and the constraint value, respectively, and *S_c_* is calculated by the original images:

(17) $$S_{c}=H(S_{I},S_{V})=\sqrt{{\left(S_{I}\right)}^{2}+{\left(S_{V}\right)}^{2}}$$

*H* represents the synthesis of the two-source image’s salient maps; we need to select the suitable area to constrain the low-frequency coefficients. *S_(I)_* represents the salient area in the infrared image and the *S_(B)_* represents the salient area in the visible image. As in Equation (17), the difference between salient and non-salient areas were more pronounced, so the salient area can be emphasized.

We calculate the fused low frequency with the constraints, as follows:

(18) $$E_{d}=\int {\left\|f_{d}-\alpha \cdot I_{d}-\beta \cdot V_{d}\right\|}_{2}^{2}+\int {\left\|S_{f_{d}}-S_{c}\right\|}_{1}$$

Discretizing this equation as:

(19) $$E_{d}=\sum_{\Omega }{\left(f_{d}-\alpha \cdot I_{d}-\beta \cdot V_{d}\right)}^{2}+\sum_{\Omega }\left|S_{f_{d}}-S_{c}\right|$$

### 2.4. Nash Equilibrium to Update the Coefficients 

After calculating the low and high coefficients, the obtained coefficients need to inverse the NSST. Before this step, we should confirm that the coefficients of the different frequencies are the best, so we use the Nash equilibrium for this purpose.

The idea of the Nash equilibrium can be shown by the following example:

The *K* represents the final set of the strategies:

(20) $$K=\left\{E_{d}^{*},E_{g}^{*}\right\}$$

We need to ensure that the final choice $E_{d}^{}\in \left\{E_{d}^{*}\right\}$ is the best option for the every option of $\left\{E_{g}^{*}\right\}$ and the final choice $E_{g}^{}\in \left\{E_{g}^{*}\right\}$ is the best option for every option of $\left\{E_{d}^{*}\right\}$. We set the two parameters to update the different frequencies’ coefficients. Using $\sigma_{d}$ to update the low-frequency coefficients, and $\sigma_{g}$ to update the high-frequency coefficients, when the values of *E_d_* and *E_g_* are smaller than the parameters, we can ensure that the result of the fused image will be better. 

Thus, we obtain the fused low-frequency coefficient, and we can obtain the fused image by the inverse NSST. The overall Algorithm 1 is described below:

**Algorithm 1. The proposed method****Input:** infrared image *I*, visible image *V***Output:** fused image *f***Step 1.**Initialize parameters, α=0.5, $\sigma_{d}=0.04$, $\sigma_{g}=0.01$**Step 2.**Decompose the *I* and *V* by NSST, get the *I_d_* , {*I_g_*} and *V_d_*, {*V_g_*}**Step 3.**Compute the *E_g_* and *E_d_* by Equation (12) and (18) Where *E_g_* < *σ_g_*, and *E_d_* < *σ_d_*, compute the *f_d_* and *f_g_* Else back to the step 3, update the α to minimize the *E_g_* and *E_d_***Step 4.**Use the *f_d_* and *f_g_* to inverse NSST to obtain the output.

**Figure 3:**
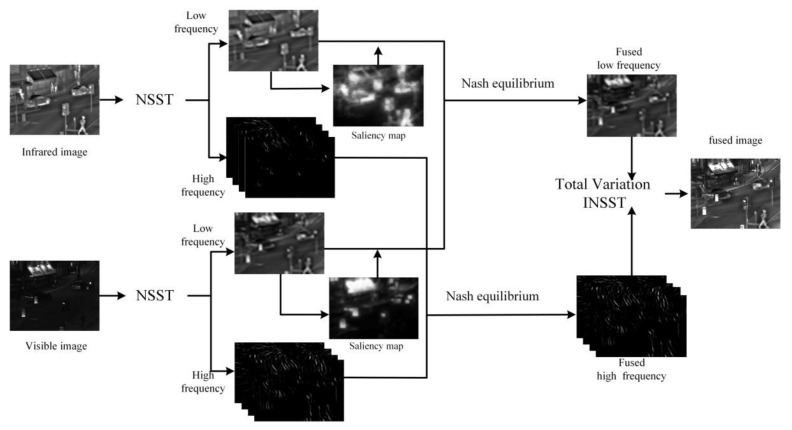
The illustration of the proposed method.

Figure 3 shows the procedure of proposed method in this paper. After the NSST transformation of the visible and infrared images, the low and high frequency coefficients were obtained. Fused the coefficients based on the Nash equilibrium principle, and the final fused image was obtained with INSST transformation.

## 3. Results and Analysis

In order to verify the effectiveness of the proposed algorithm, we experiment the infrared and visible images in MATLAB 2016a (Core i3, clocked at 3.5 GHz, memory for 8 GB). The levels of NSST is four, the directions of the first level is four, and the next level is double than the last level. There are three groups of images, which include the salient target and rich details. When compared with other algorithms, such as contrast pyramid (CP) [5], PCA [6], Guided Filter fusion (GFF) [27], saliency fusion (SAL) [7], gradient transfer and total variation (GTF) [17], non-subsampled Contourlet transform (NSCT) [11], and the NSST [15]. The validity of the algorithms is verified by subjective and objective evaluation.

**Figure 4:**
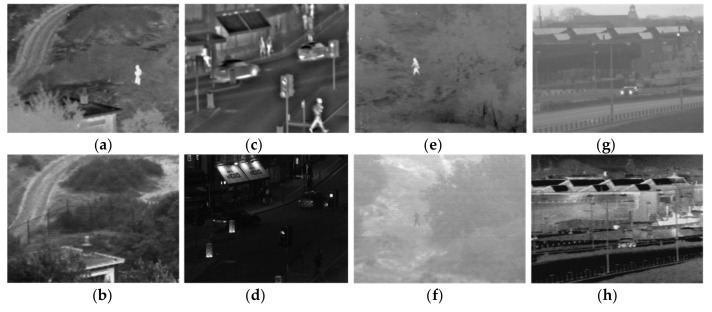
Images for experiment: (**a**,**b**) the first set of experimental images; (**c**,**d**) the second set of experimental images; (**e**,**f**) the third set of experimental images; and (**g**,**h**) the fourth set of experimental images.

In Figure 4, four sets of experimental images are registered as infrared and visible images; the first group has a more obvious salience of the target, the second set of images has rich details, and the third set of images has a salient target and a blurred background; the fourth set of images has rich details and a blurred background.

### 3.1. Performance Evaluation

**Figure 5:**
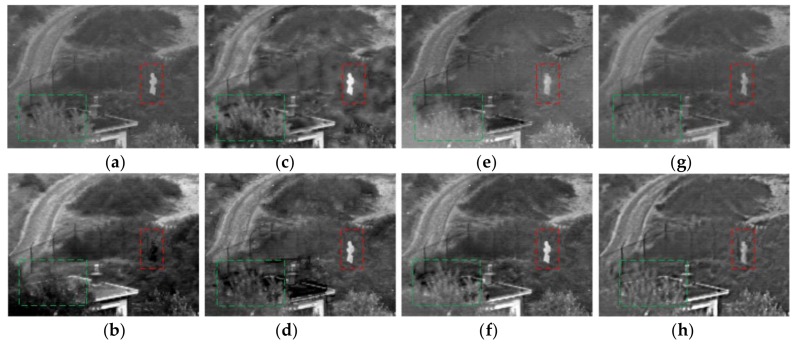
The results for first set of images. (**a**) Contrast pyramid (CP); (**b**) principal component analysis (PCA); (**c**) Guided Filter fusion (GFF); (**d**) saliency fusion (SAL); (**e**) gradient transfer and total variation (GTF); (**f**) non-subsampled Contourlet transform NSCT; (**g**) non-subsampled shearlet transform (NSST); and (**h**) proposed.

As the Figure 5 shows, analyze the red box area in the images, the result of PCA algorithm is poor, and the target is not obvious. In the Figure 5c,d,f, the target is brighter, but to enhance the target area brightness, a greater impact on the environmental area is produced at the same time. There is more serious blur in the Figure 5c, and the railing in the background is too smooth to detect in Figure 5d,f. In the images of Figure 5a,e,g, the target brightness is similar to the proposed algorithm, but the edge in Figure 5c is blurry, and the information of the background is missing. In the proposed algorithm, we reflect the target and retain the background information as much as possible. The green box area mainly reflects the details of the trees, and we can find that there is a shadow phenomenon in the result of PCA, GFF, and SAL. The results of CP, GTF, and NSCT are fuzzier. The details of the NSST has been promoted, but when compared with the proposed algorithm, our result is better in terms of the details.

**Figure 6:**
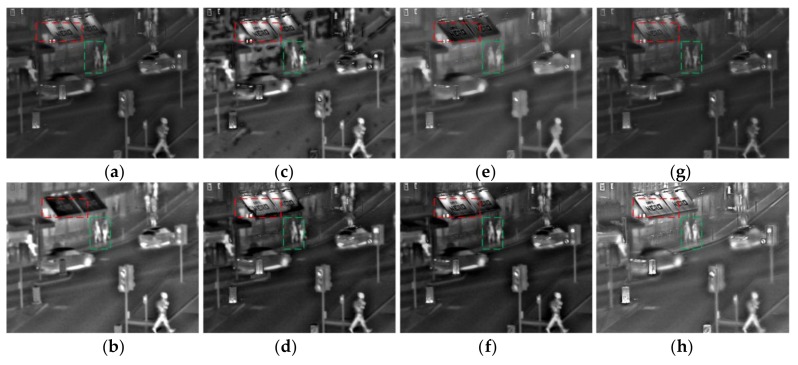
The results for second set of images. (**a**) CP; (**b**) PCA; (**c**) GFF; (**d**) SAL; (**e**) GTF; (**f**) NSCT; (**g**) NSST; and (**h**) proposed.

There are more details in Figure 6, comparing the proposed algorithm with other algorithms. The red box is the text message in the roof, low brightness around the text, and their performance is not obvious in the results of CP, PCA, GTF, and the NSST. When compared with results of GFF, SAL, and NSCT, there are no artificial artifacts in the proposed method. The proposed algorithm preserves the detailed information on the basis of maximizing the brightness of the text background, and it is more suitable for the human eye system. The green box area in the figure is a more pronounced salient target, the contrast in the CP and the NSST is insufficient, and the information of the target can be preserved in the results of PCA, SAL, and NSCT, but the seat information on the left side of the target is basically lost. The result of the GFF algorithm has black block noise near the target. The proposed algorithm is better with respect to details and salient constraints, and it is superior.

**Figure 7:**
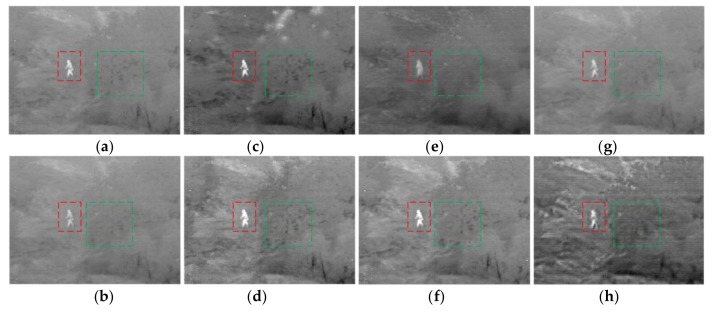
The results for third set of images. (**a**) CP; (**b**) PCA; (**c**) GFF; (**d**) SAL; (**e**) GTF; (**f**) NSCT; (**g**) NSST; and (**h**) proposed.

Figure 7 shows the results of the third set of experimental images, processed by eight algorithms, and the overall comparison shows that proposed algorithm has the best effect in terms of detail reservation. The green box area in the figure shows some of the details of the trees, there is more retention of infrared image information in the results of CP, PCA, GTF, and the NSST, but also a lack of information is preserved in the visible images. The proposed algorithm is constrained by the gradient for the high-frequency part, and the details are more reserved in the result. There are also some algorithms that preserve the details, such as GFF, SAL, and NSCT, but when compared with the proposed algorithm, they are not superior enough. The red box section is the target area, the target is vague, and the edges are affected by the artificial artifacts in the GTF’s result. Additionally, there is a loss of background information around the target in the other algorithms. The proposed algorithm can reflect the target, and it has rich information of the background at the same time. It is more conducive to further processing.

**Figure 8:**
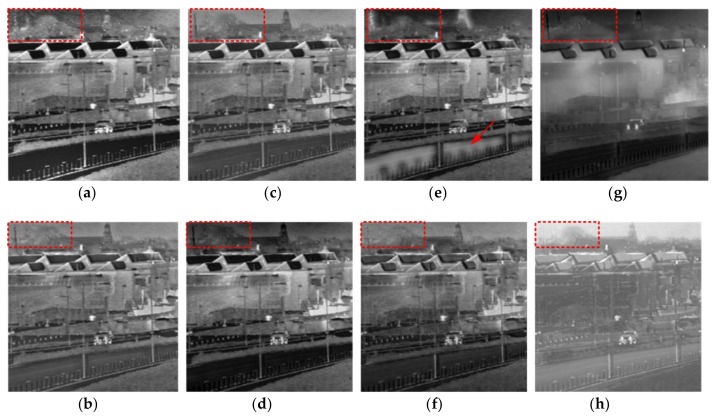
The results for fourth set of images. (**a**) CP; (**b**) PCA; (**c**) GFF; (**d**) SAL; (**e**) GTF; (**f**) NSCT; (**g**) NSST; and (**h**) proposed.

Figure 8 shows that the proposed algorithm has obvious advantages in detail handling when compared with existing algorithms. In the proposed algorithm, the high frequency coefficients are constrained by the gradient constraint, and the details are more reserved in the result. Although there are also some other algorithms that can preserve the details, such as GFF, SAL, and PCA, they are not superior enough when compared with the proposed algorithm. Observing the red box tree area, the tree target is vague and the edges are affected by the artificial artifacts in the results of NSST, GTF, and CP algorithms. The target is reflected more definitely and more details are reserved with the proposed algorithms.

### 3.2. Further Analysis

After visual comparison and the analysis of the experimental results, it is obvious that the proposed algorithm has advantages in salient target enhancement and detail reservation. The advantages and disadvantages of objective indicators can be more effective in confirming the effectiveness of the proposed algorithm. Thus, eight algorithms were evaluated by using the commonly used fusion image quality evaluation index; they are the quality of image (Q) [28], quality of edge (Qe) [29], information entropy (H), average gradient (AVG), and overall cross entropy (OCE). The results are as follows:

Table 1, Table 2, Table 3 and Table 4 list the four groups of experimental results of the quality evaluation indicators.

Through the objective data of Table 1, Table 2, Table 3 and Table 4, the numerical values of the proposed algorithm are advantageous with respect to the image quality, edge quality, and the average gradient. When comparing the information entropy with these algorithms, the proposed algorithm in the first two groups of experiments were lower than the PCA algorithm but, compared with other algorithms, the value is higher. Analyzing the PCA algorithm, the reason for the higher value is that the targets are dark in the first two experimental images, so the contrasts are poor, and so the values are higher. When comparing the evaluation of the overall cross-entropy index, the results of the proposed method are better than the other methods, and it is shown that the information obtained from the two source images is the largest. Based on the experimental results, the proposed algorithm is at the optimal level.

## 4. Conclusions

The traditional fusion algorithms are summarized in this paper, and a new fusion algorithm is proposed for the problem of large artificial parameters and large errors. The high- and low-frequency coefficients’ characteristics after the NSST are analyzed, fusing the high-frequency coefficients based on the gradient constraints, and fusing the low-frequency coefficients based on the salient constraint. Before the inverse the NSST, the Nash equilibrium is used to update the coefficient, so that the fused image can preserve the details and enhance the target at the same time. After experiments and objective analysis, the validity of the algorithm is verified.

## Figures and Tables

**Table 1:** Comparison of evaluation results of first experiment.

Algorithm	Q	Q_e_	H	AVG	OCE
CP	6.0401	55.7800	6.7564	5.3759	6.7892
PCA	5.7493	52.4211	7.4664	4.9450	6.2556
GFF	5.1017	45.3709	6.7829	4.2921	6.1763
SAL	6.0435	56.3253	7.1872	5.4797	6.4765
GTF	4.1171	35.6740	6.6778	3.4104	6.8306
NSCT	6.0186	50.6460	6.9740	4.8288	6.6516
NSST	3.4279	29.1192	6.2750	2.7837	5.9401
Proposed	6.1977	59.9885	6.6024	5.9197	6.9581

**Table 2:** Comparison of evaluation results of second experiment.

Algorithm	Q	Q_e_	H	AVG	OCE
CP	4.6699	43.5812	6.1763	4.4344	6.8412
PCA	4.1114	35.8304	6.9892	3.3226	7.1751
GFF	4.7462	41.7774	6.8365	3.9003	7.1223
SAL	4.9055	41.0244	6.8306	3.8867	7.3618
GTF	2.8752	25.9216	6.6204	2.4483	7.4233
NSCT	4.5353	38.1520	6.6516	3.5918	7.4169
NSST	2.5643	21..4194	5.9401	2.0280	7.3417
Proposed	5.0675	45.5668	6.8449	4.5225	7.4521

**Table 3:** Comparison of evaluation results of third experiment.

Algorithm	Q	Q_e_	H	AVG	OCE
CP	6.5900	36.4337	6.2311	4.2565	6.6155
PCA	2.8677	17.8803	5.9258	1.9602	6.4579
GFF	4.9492	28.1646	6.2764	3.2447	6.5692
SAL	5.3270	33.3692	6.2769	3.6519	6.7221
GTF	2.9241	21.2003	6.0205	2.1727	6.0142
NSCT	4.9417	29.5920	6.1613	3.2930	6.8353
NSST	3.1185	18.4770	5.9227	2.0706	6.9215
Proposed	5.7246	40.3332	6.6474	4.3008	7.2371

**Table 4:** Comparison of evaluation results of fourth experiment.

Algorithm	Q	Q_e_	H	AVG	OCE
CP	2.4312	62.4540	6.3764	4.5540	3.6240
PCA	3.6473	57.6553	5.9483	3.9369	2.7300
GFF	4.5772	53.543	5.4922	3.5770	2.0700
SAL	4.2346	42.4731	4.5290	1.3330	1.0230
GTF	1.8541	55.5872	6.5158	3.5109	2.3092
NSCT	3.9298	59.8357	6.1275	3.5552	3.0523
NSST	3.1185	52.2627	5.4491	3.8873	1.8936
Proposed	5.9032	65.4193	6.4327	4.5692	3.9829
